# Associations of obesity with tracheal intubation success on first attempt and adverse events in the emergency department: An analysis of the multicenter prospective observational study in Japan

**DOI:** 10.1371/journal.pone.0195938

**Published:** 2018-04-19

**Authors:** Hiromasa Yakushiji, Tadahiro Goto, Wataru Shirasaka, Yusuke Hagiwara, Hiroko Watase, Hiroshi Okamoto, Kohei Hasegawa, on behalf of the Japanese Emergency Medicine Network investigators

**Affiliations:** 1 Department of Emergency Medicine, Kishiwada Tokushukai Hospital, Osaka, Japan; 2 Department of Emergency Medicine, Massachusetts General Hospital, Boston, Massachusetts, United States of America; 3 Department of Pediatric Emergency and Critical Care Medicine, Tokyo Metropolitan Children’s Medical Center, Tokyo, Japan; 4 Department of Surgery, University of Washington, Seattle, Washington, United States of America; 5 Center for Clinical Epidemiology, St. Luke’s International University, Tokyo, Japan; 6 Harvard Medical School, Boston, Massachusetts, United States of America; McMaster University, CANADA

## Abstract

Obesity is deemed to increase the risk of difficult tracheal intubation. However, there is a dearth of research that examines the relationship of obesity with intubation success and adverse events in the emergency department (ED). We analyzed the data from a prospective, observational, multicenter study—the Japanese Emergency Airway Network (JEAN) 2 study from 2012 through 2016. We included all adults (aged ≥18 years) who underwent tracheal intubation in the ED. Patients were categorized into three groups according to their body mass index (BMI): lean (<25.0 kg/m²), overweight (25.0–29.9 kg/m²), and obesity (≥30.0 kg/m²). Outcomes of interest were intubation success on the first attempt and intubation-related adverse events. Of 6,889 patients who are eligible for the analysis, 5,370 patients (77%) were lean, 1,177 (17%) were overweight, and 342 (4%) were obese. Compared to the lean patients, the intubation success rates were significantly lower in the overweight and obese patients (70.9% in lean, 66.4% in overweight, and 59.3% in obese patients; P<0.001). In the multivariable analysis, compared to the lean patients, overweight (adjusted odds ratio [OR], 0.85; 95%CI, 0.74–0.98) and obese (adjusted OR, 0.62; 95%CI, 0.49–0.79) patients had a significantly lower success rate on the first attempt. Additionally, obesity was significantly associated with a higher risk of adverse events (adjusted OR, 1.62; 95%CI, 1.23–2.13). Based on the data from a multicenter prospectively study, obesity was associated with a lower success rate on the first intubation attempt and a higher risk of adverse event in the ED.

## Introduction

### Background

Emerging evidence indicates that difficult intubation and repeated intubation attempts are related to a higher risk of intubation-related adverse events in the emergency department (ED) [1–4]. Thus, early recognition of difficult airway with an optimal preparation and use of alternative methods is critical. The anesthesia literature has identified the factors that predicts difficult intubations—e.g., short thyromental distance, large neck circumference, and obesity [5,6].

Despite the differences in patient population and available resources from the anesthesia settings, there is insufficient existing evidence of the association between obesity and intubation outcomes in the ED. The limited emergency medicine literature—which is based on retrospective studies [7,8]—is conflicting with the intubation success rates in obese patients to be no different from [7] or higher than [8] those in non-obese patients in the ED. As obesity is a common comorbid condition in the ED population [9], further clarification of its impact on the intubation outcomes will inform the strategies to guide optimal emergency airway management in the ED.

### Objectives

To address the knowledge gap in the literature, we aimed to investigate the association of obesity with intubation success and adverse event rates in the ED, by using the data from a large prospective multicenter study. We hypothesized that obesity is associated with a lower success rate of first intubation attempt and a higher rate of adverse events in the ED.

## Materials and methods

### Study design and setting

We analyzed the data from a prospective, observational, multicenter study—the Japanese Emergency Airway Network (JEAN) 2 study—from February 2012 through November 2016. The study setting, methods of measurement, and measured variables have been reported previously [3,4,10–14]. In short, the JEAN 2—a consortium of 14 academic and community medical centers from different geographic regions across Japan—prospectively enrolled all pediatric and adult patients who underwent emergency tracheal intubation in one of the participating EDs. All 14 EDs were staffed by emergency attending physicians and had affiliations with emergency medicine residency training programs. The participating institutions included 11 Critical Medical Care Centers and had an average ED census of 31,000 patient visits per year (range 14,000 to 66,000). In this observational study, each ED maintained individual protocols about the procedures and policy for ED airway management. Intubations were performed by attending physicians, or by resident physicians at the discretion of attending physicians. The study was approved by the Institutional Review Board of each participating center, including the Institutional Review Board of Kishiwada Tokushukai Hospital, with waiver of informed consent prior to data collection.

### Selection of participants

For the present analysis, we included all adult patients (aged ≥18 years) who underwent intubation in one of the participating EDs during a 58-month period (from February 2012 through November 2016).

### Data collection

The JEAN 2 study prospectively collected the data for consecutive patients. After each intubation, the intubator—physician performing each intubation—completed a standardized data collection form that included the patient’s age, sex, estimated weight and height, primary indication for intubation, methods and medications of airway management, devices used to facilitate the intubation, level of training and specialty of the intubator, number of attempts, success or failure at each attempt, vital signs, and intubation-related adverse events. We monitored compliance with data form completion. Where the data collection form was missing, we interviewed the involved physicians and reviewed medical records to ascertain the airway management details. These *post-hoc* interviews occurred within two weeks of the patient encounter.

### Primary exposure

The primary exposure of interest was the patient obesity status, according to patient’s body mass index (BMI): lean (<25.0 kg/m²), overweight (25.0–29.9 kg/m²), and obesity (≥30.0 kg/m²) [7,8]. BMI was calculated based on the weight and height that are estimated by the intubator at the intubation in the ED. The literature has indicated that the physician-estimated weight, height, and BMI category are relatively accurate [15,16].

### Outcome measures

The outcomes of interest were intubation success on the first attempt and intubation-related adverse events. An “intubation attempt” was defined as a single insertion of the device (direct or video laryngoscope, regardless of video channeling or use of adjunct devices) past the teeth. An attempt was successful if it resulted in an endotracheal tube being placed through the vocal cords. Intubation-related adverse events included cardiac arrest, post-intubation hypoxemia (pulse oximetry saturation <90%), hypotension (systolic blood pressure <90 mmHg), dysrhythmia, esophageal intubation with delayed recognition, mainstem bronchial intubation, airway trauma, dental or lip trauma, regurgitation, and allergic reaction [3,4,10–14]. Esophageal intubation was diagnosed with physical examination, ultrasonography, end-tidal CO_2_ monitor, chest x-ray, or any combination of these methods.

### Statistical analysis

First, we compared the patient characteristics between the BMI categories by using χ^2^ or Kruskal-Wallis tests as appropriate. Next, to determine the association of BMI category with each of the intubation outcomes, we constructed multivariable random-effects models with binary response to account for patient clustering within the EDs. The models adjusted for potential confounders, including age, sex, primary indication for intubation (medical cardiac arrest, traumatic cardiac arrest, medical non-cardiac-arrest, and traumatic non-cardiac-arrest), methods of intubation (no medication, rapid sequence intubation [RSI], sedative only, and others), intubation devices (direct laryngoscope, video laryngoscope, and others), and training level and specialty of intubator (transitional-year residents [post-graduate year 1 and 2 physicians], emergency medicine residents, emergency physician, and others). In the sensitivity analysis, we repeated the analyses with stratification by cardiac arrest as the primary indication. We also repeated the subgroup analysis in the patients who underwent RSI. P-values of <0.05 were regarded as statistically significant. All statistical analyses were performed using STATA 14.1 (StataCorp, College Station, TX).

## Results

### Baseline characteristics of patients

We recorded 7,657 patients (capture rate, 97%; S1 Fig) who underwent emergency airway management during the 58-month study period. We excluded 252 pediatric patients (aged <18 years) or without the information on age, 457 patients without the information on BMI, and 59 patients who underwent surgical intubation. Of 6,889 patients eligible for the analysis, 5,370 patients (77%) were lean, 1,177 (17%) were overweight, and 342 (4%) were obese. Baseline characteristics of these groups are shown in Table 1. Compared to lean patients, overweight and obese patients were younger and more likely to be intubated for medical indication, intubated with RSI, and intubated with a video laryngoscope (all P<0.001).

**Table 1:** Baseline characteristics of 6,889 patients who underwent tracheal intubation in the emergency department, according to body mass index category.

Variables	Body mass index (kg/m^2^) category			P value
	<25.0 (Lean) n = 5,370	25.0–29.9 (Overweight) n = 1,177	≥30.0 (Obesity) n = 342	
Age, year, median (IQR)	73 (60–82)	66 (53–76)	60 (45–80)	<0.001
Male	3,322 (61)	784 (66)	171 (50)	<0.001
Primary indication				<0.001
Medical arrest	2,045 (38)	377 (32)	100 (29)	
Traumatic arrest	219 (4)	45 (4)	68 (2)	
Medical indication	2,475 (46)	606 (51)	209 (61)	
Traumatic indication	631 (11)	149 (12)	25 (7)	
Methods				<0.001
No medication	3,096 (57)	573 (48)	161 (47)	
Rapid sequence intubation	1,530 (28)	388 (32)	114 (33)	
Sedative only	539 (10)	149 (12)	51 (14)	
Others*	205 (3)	67 (4)	16 (4)	
Devices				<0.001
Direct laryngoscope	3,889 (72)	833 (70)	223 (65)	
Video laryngoscope	1,369 (25)	307 (26)	100 (29)	
Others†	112 (2)	37 (3)	19 (5)	
Training level and specialty of intubator				0.46
Emergency physician	983 (18)	246 (20)	66 (19)	
Emergency medicine resident	1,535 (28)	338 (28)	97 (28)	
Transitional year resident‡	2,383 (44)	495 (42)	145 (42)	
Others§	469 (8)	98 (8)	34 (9)	

Abbreviation: IQR, interquartile range. Data were presented as number (percentage) of patients unless otherwise indicated.

*Others include intubations using paralytics or analgesics only

^†^Others include intubation using a bougie or fiberoptic scope

^‡^Defined as post-graduate years 1 and 2

^§^Others include intbuations by surgeon and anesthesiologist

### Associations between BMI category and first-pass intubation success rate

**Fig 1:**
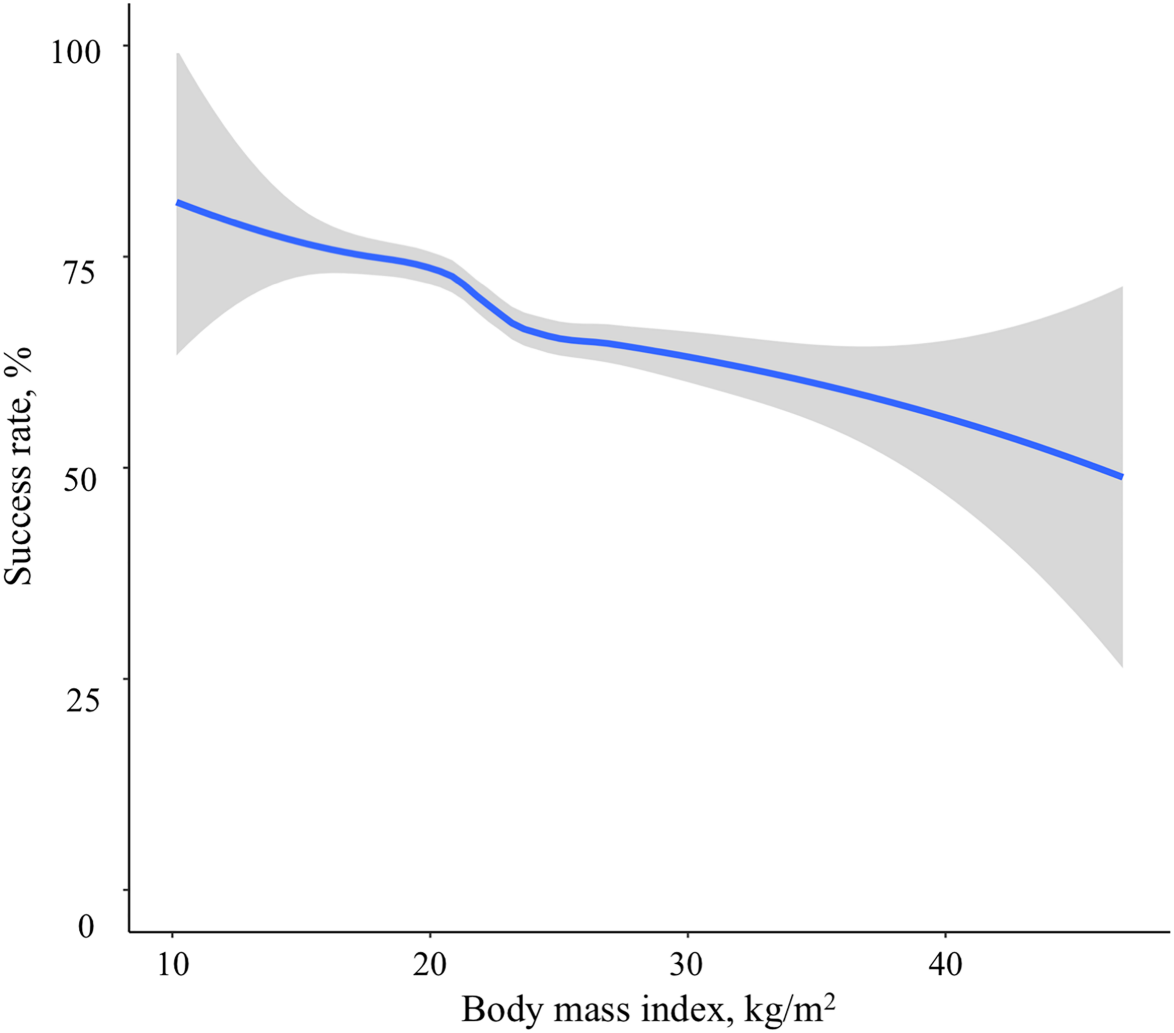
Association of body mass index with the success rate on the first intubation attempt. The fitted line represents locally weighted scatterplot smoothed (LOWESS) curve. There was a negative relationship between body mass index and success rate on the first intubation attempt.

Overall, there was a negative relationship between BMI and success rate on the first intubation attempt (Fig 1). The success rates were 70.9% (95%CI, 69.7%-72.1%) in lean patients, 66.4% (95%CI, 63.7%-69.1%) in overweight patients, and 59.3% (95%CI, 53.9%-64.6%) in obese patients (Table 2). In the unadjusted model, compared to lean patients, overweight and obesity patients had a significantly lower success rate on the first intubation attempt (unadjusted OR for overweight 0.85 [95%CI 0.74–0.97] P = 0.02; unadjusted OR for obesity 0.62 [95%CI 0.49–0.78] P<0.001). In the multivariable model adjusting for age, sex, primary indication for intubation, methods of intubation, devices for intubation, and training level and specialty of the intubator, these associations remained significant (adjusted OR for overweight 0.85 [95%CI 0.74–0.98] P = 0.03; adjusted OR for obesity 0.62 [95%CI 0.49–0.79] P<0.001). While there were some differences in the success rates between the training level and specialty groups (S1 Table), the associations remained significant (Table 2).

**Table 2:** Unadjusted and adjusted associations between body mass index and success rates on the first intubation attempt.

Body mass index category	Success rate (number of successes/number of first attempts)	Unadjusted OR (95% CI)	P value	Adjusted OR* (95% CI)	P value
Lean	70.9% (3,808/5,370)	Reference		Reference	
Overweight	66.4% (782/1,177)	0.85 (0.74–0.97)	0.02	0.85 (0.74–0.98)	0.03
Obesity	59.3% (203/342)	0.62 (0.49–0.78)	<0.001	0.62 (0.49–0.79)	<0.001

Abbreviations: OR, odds ratio; CI, confidence interval

*Adjusted for age, sex, primary indication for intubation, methods of intubation, devices for intubation, and training level and specialty of the intubator

### Associations between BMI category and intubation-related adverse event rates

**Fig 2:**
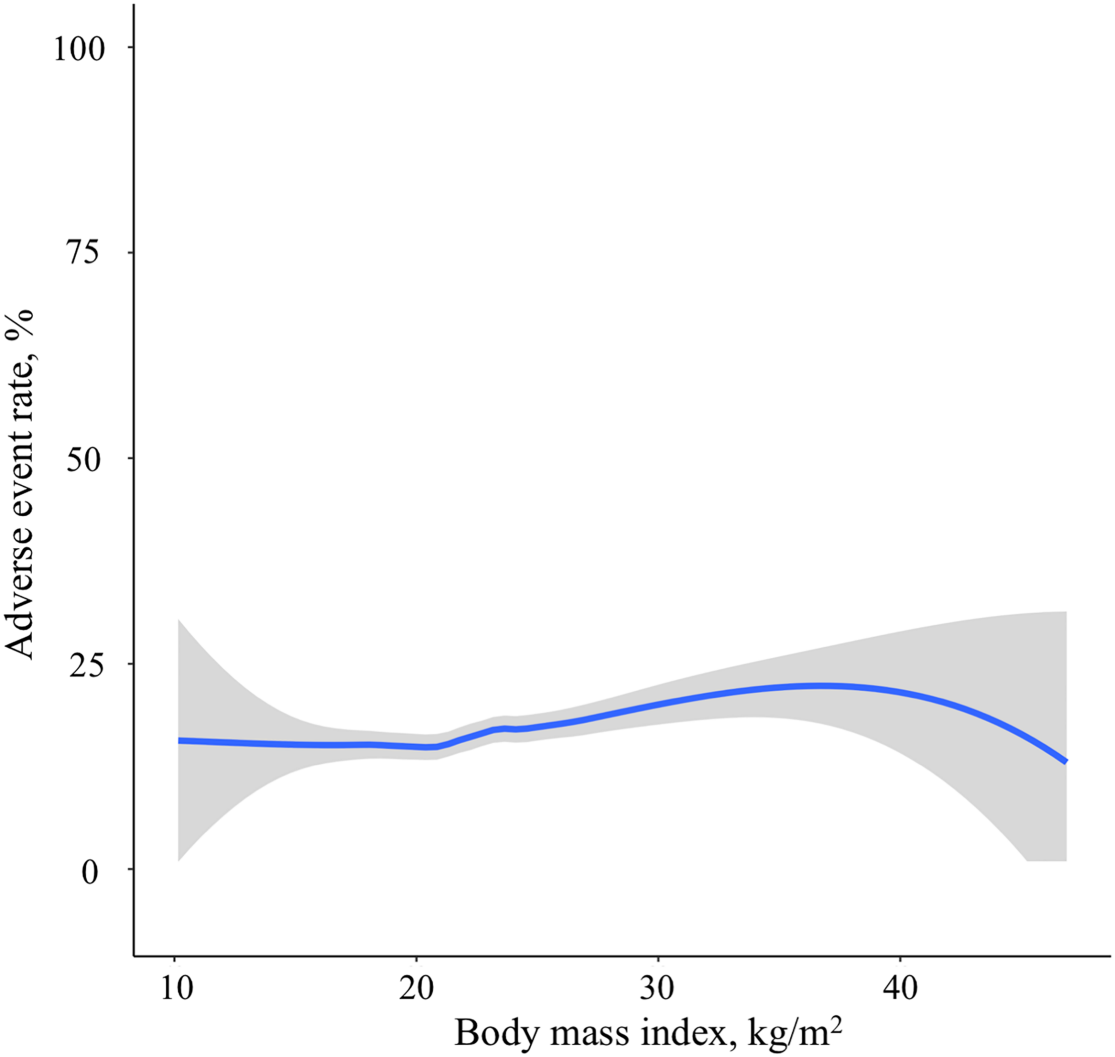
Association of body mass index with the adverse event rates. The fitted line represents locally weighted scatterplot smoothed (LOWESS) curve. There was a positive relationship between body mass index and adverse event rates.

There was a positive relationship between BMI and adverse event rates (Fig 2). Table 3 describes the unadjusted and adjusted associations between BMI category and adverse event rates. The adverse event rates were 15.8% (95%CI, 14.9%-16.9%) in lean patients, 18.1% (95%CI, 15.9%-20.4%) in overweight patients, and 24.2% (95%CI, 19.8%-29.2%) in obese patients. In the both unadjusted and adjusted models, obesity was significantly associated with a higher risk of adverse events (unadjusted OR 1.61 [95%CI 1.24–2.10] P<0.001; adjusted OR 1.62 [95%CI 1.23–2.13] P<0.001).

**Table 3:** Unadjusted and adjusted associations between body mass index and intubation-related adverse events.

Body mass index category	Adverse event rates (number of adverse events / number of attempts)	Unadjusted OR (95% CI)	P value	Adjusted OR* (95% CI)	P value
Lean	15.8% (852/5,370)	Reference		Reference	
Overweight	18.1% (213/1,177)	1.12 (0.94–1.32)	0.17	1.13 (0.95–1.35)	0.13
Obesity	24.2% (83/342)	1.61 (1.24–2.10)	<0.001	1.62 (1.23–2.13)	<0.001

Abbreviations: OR, odds ratio; CI, confidence interval

* Adjusted for age, sex, primary indication for intubation, methods of intubation, devices for intubation, and training level and specialty of the intubator.

### Sensitivity analyses

To assess the robustness of the results, we performed a series of sensitivity analyses. In the stratified analysis, in both cardiac arrest and non-cardiac-arrest patients, obese patients had a significantly lower success rate (adjusted OR 0.59 [95%CI 0.39–0.90] P = 0.01 in cardiac arrest; adjusted OR 0.64 [95%CI 0.47–0.86] P = 0.003 in non-cardiac-arrest; S2 Table) compared to lean patients. Additionally, obesity was associated with a significantly higher risk of adverse events (adjusted OR 1.62 [95%CI 1.00–2.93] P = 0.04 in cardiac arrest; adjusted OR 1.52 [95%CI 1.10–2.08] P = 0.009 in non-cardiac-arrest; S3 Table). Among the patients who underwent RSI, obesity was associated with a significantly lower success rate (adjusted OR 0.59 [95%CI 0.39–0.90] P = 0.03; S4 Table). With the limited statistical power in this subgroup analysis, obese patients also had a non-significantly higher rate of adverse event (adjusted OR 1.40 [95%CI 0.88–2.22] P = 0.15; S5 Table). An exploratory analysis of the relationship between obesity and esophageal intubations did not demonstrate significant associations (all P>0.05; S6 Table).

## Discussion

In this large multicenter prospective study of 6,889 patients who underwent emergency airway management in Japan, we found that overweight and obesity were significantly associated with a lower success rate on the first intubation attempt in the ED even with adjustment for potential confounders. In addition, obesity was also associated with a higher rate of adverse events in the ED. These significant associations persisted across different patient subgroups.

Our findings are consistent with the previous literature that demonstrated the association between obesity and decreased intubation success rates in the operating room and ED settings [6,8,17]. For example, in a prospective study of intubations in the operating room, overweight and obesity were associated with a higher risk of failure on the first and second intubation attempts [6]. Another retrospective study of 1,053 intubations at a single academic ED reported that, compared with lean and overweight patients, obese patients were more likely to require multiple intubation attempts [8]. However, few other studies reported inconsistent findings—no significant association between obesity and intubation success rate [7,18]. The apparent inconsistency across the studies may be attributable to the differences in the study design, setting (e.g., single center study), population, and outcomes (e.g., intubation success within three or more attempts, intubation difficulty scale scores, or Cormack score) [7,8,17,18]. Instead, given the emerging evidence on the importance of first-pass intubation success (e.g., its contribution to the decreased rate of adverse events) [1–4], the current study has focused on this clinically important outcome in the ED.

We also found the significant association between obesity and a higher risk of intubation-related adverse events. The sparse literature has investigated this association in the ED setting [7,8]. For example, a retrospective single-center study of 1,435 ED intubations reported a statistically significant but clinically non-significant higher rate of early respiratory complications [7]. Another retrospective single-center study of 1,053 ED intubations reported that, compared to lean patients, overweight and obese patients are more likely to have immediate post-intubation complications [8]. Our multicenter study—with a sample size that are many times larger than any other prior ED studies on this topic [7,8]–builds on their findings and extends them by demonstrating the robust association of obesity with adverse event rates in addition to that with first-pass success rates, clinically important outcomes in the ED [1–4].

The underlying mechanisms of the observed associations are likely multifactorial. For example, the link between obesity and lower intubation success rates may be explained by suboptimal medication dosing (sedatives and neuromuscular blockades), altered upper airway anatomy, reduced glottic visualization, or any combination of these factors. Indeed, the literature has documented that obese individuals have excessive soft tissues in the velopalate, retropharynx, and submandibular regions and that these excessive tissues contribute to difficulty in intubation [18,19]. Furthermore, the link between obesity and higher adverse event rates may be attributable to the higher likelihood of repeated intubation attempts [1–4], reduced tidal and expiratory reserve volumes [20], lower functional residual capacity [21], excess soft tissues in the airway [19], and changes in the upper airway and fat mass on the chest wall leading to difficult mask ventilation [22]. Further investigation into the underlying mechanisms would inform the development of optimal airway management strategies in this high-risk population.

### Potential limitations

We acknowledge that this study has several potential limitations. First, in the current study population, only 17% were overweight and 4% were obese (BMI ≥30.0 kg/m^2^), which are lower than the previous reports in other industrialized countries [7,8]. Second, the BMI was calculated based on the weight and height estimated by the intubator, and hence misclassification of the BMI category is possible. However, prior study indicated that the physician-estimated weight and height as well as BMI are relatively accurate [15,16]. Furthermore, in the emergency setting, the exact patient’s height and weight are often unknown and the use of objective measurements with calibrated instruments are not feasible. Therefore, our study reflects the ED airway management in the real-world setting and has implications on the clinical decision-making in the ED. Third, our study did not have the information of pre-intubation techniques (e.g., positioning, passive oxygenation, sedation for first look prior to paralysis). Fourth, as with any observational studies, the associations of obesity with intubation outcomes does not necessarily prove causality and might be confounded by unmeasured factors (e.g., individual intubator experience). Finally, our study sample chiefly consisted of academic EDs in Japan. While it is tempting to dismiss the generalizability of these inferences, the observed associations between obesity and intubation-related outcomes persisted across several analytical assumptions. Furthermore, multiple studies arrived at similar conclusions despite the different patient populations (e.g., operating room populations [6,17]) and healthcare setting [8].

## Conclusions

Based on the data from a large, prospective, multicenter study of 6,889 ED intubations, we found that obesity were significantly associated with a lower success rate on the first intubation attempt. In addition, obesity was also associated with a higher risk of intubation-related adverse events. For clinicians, our data underscore the importance of early recognitions of markers for difficult intubation (e.g., obesity) and optimization (e.g., optimal positioning). Lastly, for researchers, our findings should facilitate further investigation into the development of effective airway management measures in this high-risk population.

## Supporting information

S1 Fig Patients receiving emergency airway management in the emergency department. (TIF)

S1 Table Success rate on the first intubation attempt according to the training level and specialty of intubator. (DOCX)

S2 Table Unadjusted and adjusted associations between body mass index and success rates on the first intubation attempt with stratification by cardiac arrest as the primary indication. (DOCX)

S3 Table Unadjusted and adjusted associations between body mass index and intubation-related adverse events with stratification by cardiac arrest as the primary indication. (DOCX)

S4 Table Unadjusted and adjusted associations between body mass index and success rates on the first intubation attempt in patients who underwent rapid sequence intubation. (DOCX)

S5 Table Unadjusted and adjusted associations between body mass index and intubation-related adverse event in patients who underwent rapid sequence intubation. (DOCX)

S6 Table Unadjusted and adjusted associations between body mass index and esophageal intubation. (DOCX)
