# Supplementary material for: Associations of obesity with tracheal intubation success on first attempt and adverse events in the emergency department: An analysis of the multicenter prospective observational study in Japan
Source: PLoS One. 2018 Apr 19;13(4):e0195938. doi: 10.1371/journal.pone.0195938 (PMC5908180; doi:10.1371/journal.pone.0195938)
Supplement: S1 Table — (DOCX) [file pone.0195938.s002.docx]

**S1 Table. Success rate on the first intubation attempt according to the training level and specialty of intubator.**

| **body mass index category** | **Success rate on the first intubation attempt**  (number of success / number of attempts) | | | |
| --- | --- | --- | --- | --- |
|  | **Transitional year residents*** | **Emergency medicine residents** | **Emergency**  **physicians** | **Other**  **specialties†** |
| Lean | 62.3% (1,484/2,383) | 78.5% (1,205/1,535) | 82.0% (806/983) | 66.7% (313/469) |
| Overweight | 57.4% (284/495) | 70.7% (239/338) | 77.6% (191/246) | 69.4% (68/98) |
| Obesity | 44.8% (65/145) | 67.0% (65/97) | 77.3% (51/66) | 64.7% (22/34) |

***** Post-graduate year 1 and 2 physicians

† Surgeon and anesthesiologist
