# Supplementary material for: Associations of obesity with tracheal intubation success on first attempt and adverse events in the emergency department: An analysis of the multicenter prospective observational study in Japan
Source: PLoS One. 2018 Apr 19;13(4):e0195938. doi: 10.1371/journal.pone.0195938 (PMC5908180; doi:10.1371/journal.pone.0195938)
Supplement: S2 Table — (DOCX) [file pone.0195938.s003.docx]

**S2 Table.** **Unadjusted and adjusted associations between body mass index and success rates on the first intubation attempt with stratification by cardiac arrest as the primary indication.**

| **Indication** | **BMI category** | **Success rates**  (number of successes/number of first attempts) | **Unadjusted OR**  (95% CI) | **P value** | **Adjusted OR***  (95% CI) | **P value** |
| --- | --- | --- | --- | --- | --- | --- |
| Cardiac arrest | Lean | 71.9%  (1,628/2,264) | Reference |  | Reference |  |
|  | Overweight | 67.3%  (284/422) | 0.88  (0.70-1.10) | 0.27 | 0.90  (0.71-1.14) | 0.38 |
|  | Obesity | 54.6%  (59/108) | 0.54  (0.36-0.81) | 0.003 | 0.59  (0.39-0.90) | 0.01 |
| Non-cardiac-arrest | Lean | 70.0%  (2,180/3,106) | Reference |  | Reference |  |
|  | Overweight | 65.9%  (498/755) | 0.83  (0.70-0.99) | 0.04 | 0.83  (0.69-1.00) | 0.05 |
|  | Obesity | 61.5%  (144/234) | 0.65  (0.49-0.86) | 0.003 | 0.64  (0.47-0.86) | 0.003 |

Abbreviations: BMI, body mass index; OR, odds ratio; CI, confidence interval

* Adjusted for age, sex, primary indication for intubation (medical vs. trauma), methods of intubation, devices for intubation, and training level and specialty of the intubator.
