# Supplementary material for: Associations of obesity with tracheal intubation success on first attempt and adverse events in the emergency department: An analysis of the multicenter prospective observational study in Japan
Source: PLoS One. 2018 Apr 19;13(4):e0195938. doi: 10.1371/journal.pone.0195938 (PMC5908180; doi:10.1371/journal.pone.0195938)
Supplement: S3 Table — (DOCX) [file pone.0195938.s004.docx]

**S3 Table. Unadjusted and adjusted associations between body mass index and intubation-related adverse events with stratification by cardiac arrest as the primary indication.**

| **Indication** | **BMI category** | **Adverse event rates**  (number of adverse events / number of attempts) | **Unadjusted OR**  (95% CI) | **P value** | **Adjusted OR***  (95% CI) | **P value** |
| --- | --- | --- | --- | --- | --- | --- |
| Cardiac arrest | Lean | 9.3%  (212/2,264) | Reference |  | Reference |  |
|  | Overweight | 9.9%  (42/422) | 0.95  (0.66-1.36) | 0.79 | 1.13  (0.70-1.46) | 0.93 |
|  | Obesity | 19.4%  (21/108) | 1.90  (1.14-3.18) | 0.01 | 1.62  (1.00-2.93) | 0.04 |
| Non-cardiac- arrest | Lean | 20.6%  (640/3,106) | Reference |  | Reference |  |
|  | Overweight | 22.6%  (171/755) | 1.16  (0.95-1.41) | 0.13 | 1.19  (0.98-1.45) | 0.07 |
|  | Obesity | 26.5%  (62/234) | 1.44  (1.06-1.96) | 0.01 | 1.52  (1.10-2.08) | 0.009 |

Abbreviations: BMI, body mass index; OR, odds ratio; CI, confidence interval

* Adjusted for age, sex, primary indication for intubation (medical vs. trauma), methods of intubation, devices for intubation, and training level and specialty of the intubator
