# Supplementary material for: Associations of obesity with tracheal intubation success on first attempt and adverse events in the emergency department: An analysis of the multicenter prospective observational study in Japan
Source: PLoS One. 2018 Apr 19;13(4):e0195938. doi: 10.1371/journal.pone.0195938 (PMC5908180; doi:10.1371/journal.pone.0195938)
Supplement: S4 Table — (DOCX) [file pone.0195938.s005.docx]

**S4 Table. Unadjusted and adjusted associations between body mass index and success rates on the first intubation attempt in patients who underwent rapid sequence intubation.**

| **BMI category** | **Success rates**  (number of successes/number of first attempts) | **Unadjusted OR**  (95% CI) | **P value** | **Adjusted OR***  (95% CI) | **P value** |
| --- | --- | --- | --- | --- | --- |
| Lean | 77.5%  (1,186/1,530) | Reference |  | Reference |  |
| Overweight | 73.4%  (285/388) | 0.88  (0.70-1.10) | 0.21 | 0.90  (0.71-1.14) | 0.14 |
| Obesity | 71.0%  (81/114) | 0.54  (0.36-0.81) | 0.09 | 0.59  (0.39-0.90) | 0.03 |

Abbreviations: BMI, body mass index; OR, odds ratio; CI, confidence interval

* Adjusted for age, sex, primary indication for intubation, devices for intubation, and training level and specialty of the intubator.
