# Supplementary material for: Associations of obesity with tracheal intubation success on first attempt and adverse events in the emergency department: An analysis of the multicenter prospective observational study in Japan
Source: PLoS One. 2018 Apr 19;13(4):e0195938. doi: 10.1371/journal.pone.0195938 (PMC5908180; doi:10.1371/journal.pone.0195938)
Supplement: S5 Table — (DOCX) [file pone.0195938.s006.docx]

**S5 Table. Unadjusted and adjusted associations between body mass index and intubation-related adverse event in patients who underwent rapid sequence intubation.**

| **BMI category** | **Adverse event rates**  (number of adverse events / number of attempts) | | **Unadjusted OR**  (95% CI) | **P value** | **Adjusted OR***  (95% CI) | **P value** |
| --- | --- | --- | --- | --- | --- | --- |
| Lean | | 21.1%  (324/1,530) | Reference |  | Reference |  |
| Overweight | | 24.2%  (94/388) | 1.24  (0.95-1.63) | 0.10 | 1.30  (0.99-1.71) | 0.05 |
| Obesity | | 24.5%  (28/114) | 1.24  (0.79-1.95) | 0.33 | 1.40  (0.88-2.22) | 0.15 |

Abbreviations: BMI, body mass index; OR, odds ratio; CI, confidence interval

* Adjusted for age, sex, primary indication for intubation, devices for intubation, and training level and specialty of the intubator
