# Supplementary material for: Associations of obesity with tracheal intubation success on first attempt and adverse events in the emergency department: An analysis of the multicenter prospective observational study in Japan
Source: PLoS One. 2018 Apr 19;13(4):e0195938. doi: 10.1371/journal.pone.0195938 (PMC5908180; doi:10.1371/journal.pone.0195938)
Supplement: S6 Table — (DOCX) [file pone.0195938.s007.docx]

**S6 Table. Unadjusted and adjusted associations between body mass index and esophageal intubation**.

| **Body mass index category** | **Adverse event rates**  (number of adverse events / number of attempts) | **Unadjusted OR**  (95% CI) | **P value** | **Adjusted OR***  (95% CI) | **P value** |
| --- | --- | --- | --- | --- | --- |
| Lean | 4.4% (238/5,370) | Reference |  | Reference |  |
| Overweight | 3.9% (46/1,177) | 0.84 (0.60-1.16) | 0.28 | 0.85 (0.61-1.18) | 0.34 |
| Obesity | 5.2% (18/342) | 1.15 (0.70-1.90) | 0.58 | 1.16 (0.69-1.93) | 0.58 |

Abbreviations: OR, odds ratio; CI, confidence interval

* Adjusted for age, sex, primary indication for intubation, methods of intubation, devices for intubation, and training level and specialty of intubator
